# Fast Screening of Whole Blood and Tumor Tissue for Bladder Cancer Biomarkers Using Stochastic Needle Sensors

**DOI:** 10.3390/s20082420

**Published:** 2020-04-24

**Authors:** Raluca-Ioana Stefan-van Staden, Damaris-Cristina Gheorghe, Viorel Jinga, Cristian Sorin Sima, Marius Geanta

**Affiliations:** 1 Laboratory of Electrochemistry and PATLAB, National Institute of Research for Electrochemistry and Condensed Matter, 202 Splaiul Independentei Street, 060021 Bucharest-6, Romania; gheorghe.damaris16@gmail.com; 2 Faculty of Medicine, University of Medicine and Pharmacy ”Carol Davila”, 050474 Bucharest, Romania; vioreljinga@yahoo.com (V.J.); cristian.sima88@gmail.com (C.S.S.); marius.geanta@me.com (M.G.)

**Keywords:** stochastic needle sensor, bladder cancer, p53, E-cadherin, bladder tumor antigen, hyaluronic acid

## Abstract

Bladder cancer is one of the most common urologic malignancies, which is more frequent in men than in women. The early diagnosis for this type of cancer still remains a challenge, therefore, the development of a fast screening test for whole blood and tumor tissue samples may save lives. Four biomarkers, p53, E-cadherin, bladder tumor antigen (BTA), and hyaluronic acid were considered for the screening tests using stochastic needle sensors. Three stochastic needle sensors, based on graphite powder and modified with three types of chitosan, were designed and characterized for the screening test. The proposed sensors showed low limits of quantification, and high sensitivity and selectivity levels. The recoveries of p53, E-cadherin, BTA, and hyaluronic acid in whole blood samples and tissue samples were higher than 95.00% with a relative standard deviation lower than 1.00%.

## 1. Introduction

Bladder cancer (BC) is a urothelial neoplasm and can be represented by a broad spectrum of disorders with vastly different treatment pathways, ranging from papillary (Ta) grade I tumor all the way to a highly aggressive and heterogeneous disease like a muscle invasive grade II tumor. It is the most common malignancy of the urinary tract [1], the sixth most commonly occurring cancer in men, and the seventeenth most commonly occurring cancer in women [2]. There were 550,000 new cases in 2018 [2]. Lebanon had the highest rate of bladder cancer in 2018, followed by Greece [2]. Factors that may favor the development of bladder cancer are tobacco smoke, exposure to chemical compounds such as those found in the chemical and rubber industries [3], genetic susceptibility [4], occupational risk, dietary factors, environmental pollution, and medical conditions [5].

p53 is a very important marker for cancer diagnosis and treatment [6,7]. p53 is the most investigated molecular marker in cancers; changes in p53 have been used to predict bladder cancer early detection, recurrence, progression, and mortality. Lower levels of 12.0 pg/mL predict bladder cancer. E-cadherin has a high importance in cell–cell adhesion in epithelial tissues: immunohistochemistry expression and its levels in blood have been linked to tumor aggression; and lower levels than 15 pg/mL are associated with bladder cancer [8,9]. BTA is the bladder tumor antigen and is used to detect BC from urine samples. Tests such as urine dipstick, cystoscopy, and urine cytology have been replaced in the first instance by the BTA stat test for the diagnosis of BC; pg/mL levels to ng/mL levels in whole blood are associated with bladder cancer [10]. Lokeshwar et al. stated in their paper that concentrations of hyaluronic acid are elevated in several tumors [11] including cancers of the bladder, prostate, lung, colon, intestine, and breast. An elevated value is also found in the tumor tissues and urine of bladder carcinoma patients where the sensitivity associated with bladder cancer detection (91.00%) is high; the specific range for bladder cancer was 170–500 pg/mL. The selection of biomarkers for bladder cancer was undertaken by specialists in bladder cancer, according to the statistics available, as our scope was focused on the development of a new and fast method of screening for these biomarkers, which can be found in biological fluids as well as in tumoral tissues, and not to establish if they were the best to be used in the early detection of bladder cancer.

Stochastic sensors are the only sensors able to perform qualitative and quantitative analysis. These are used for the biomedical analysis of biomarkers such as HER1, HER2, interleukins, CA19-9 [12,13,14], and their mechanism of signal development has been investigated on several occasions [12,13,14,15,16,17,18].

To our knowledge, for the assay of the proposed biomarkers, the best methods recorded to date are histochemistry for the qualitative assay of p53 [19], amperometry using an immunosensor [20], and a magnetoimmunosensor [21] for the quantitative assay of p53 [20], for E-cadherin, it is amperometry using an immunosensor [22], for BTA it is an immunoassay [23], while for the assay of hyaluronic acid, the best results were obtained using a colorimetric enzyme-coupled technique [24]. None of these methods can be applied for the simultaneous assay of the four biomarkers, and these methods cannot be applied for in vivo analysis of the biomarkers in biological fluids or tumoral tissue samples. The in vivo measurements may be needed for continuous monitoring of patients with high risk of developing this type of cancer or with risk of metastasis, or for monitoring the efficiency of the treatment, and therefore, there is a need to have needle type sensors that can be used for in vivo continuous monitoring.

In this paper, we proposed three new stochastic needle sensors based on graphite paste, modified with three types of chitosan (chitosan I (n = 371–744), chitosan II (n = 682–930), and chitosan III (n = 868–1365)) for the pattern recognition of p53, E-cadherin, BTA, and hyaluronic acid in whole blood and tissue samples with high sensitivity. The materials used for the design of the stochastic sensors were chosen for the following reasons: (1) graphite and chitosan are biocompatible materials (essential if one would like to perform in vivo analysis at a later stage), and (2) chitosan has the pores needed for the development of a signal specific for stochastic sensors. The novelty of this paper is the design of the stochastic sensors as well as the simultaneous recognition and analysis of p53, E-cadherin, BTA, and hyaluronic acid in biological samples.

## 2. Materials and Methods

### 2.1. Chemicals

All chemicals were of analytical grade. p53, E-cadherin, BTA, hyaluronic acid, graphite powder, chitosan I (n = 371–744), chitosan II (n = 682–930), chitosan III (n = 868–1365), and dimethyl sulfoxide (DMSO) were purchased from Sigma Aldrich (Milwaukee, USA). The paraffin oil was purchased from Fluka (Buchs, Switzerland).

For each analyte, standard solutions were used in order to determine the biomarkers of interest. The dilutions containing p53 (concentration range between 1.17 × 10^−15^–7.14 µg/mL), E-cadherin (concentration range between 6.50 × 10^−15^–20.00 µg/mL) and hyaluronic acid (concentration range between 3.00 × 10^−15^–0.30 mg/mL) were prepared using a phosphate buffer solution (PBS) that had the same pH value (pH = 7.5) and the dilutions containing BTA (concentration range between 2.00 × 10^−14^–0.20 mg/mL) were prepared using DMSO. An wide concentration range was selected for each biomarker in order to be able to see if the proposed sensors could be used for the early and late diagnosis of bladder cancer.

### 2.2. Instruments

For all measurements, a potentiostat/galvanostat AUTOLAB/PGSTAT 302 (Methrom, Utrecht, The Netherland) connected to a personal computer with GPES software installed was used. An electrochemical cell containing a three electrode system was also employed. The three electrode system was made out of the working electrode, which is the proposed stochastic needle sensor; the reference electrode, also known as the Ag/AgCl electrode; and the counter electrode, represented by a platinum wire.

### 2.3. Design of the Stochastic Needle Sensors

**Scheme 1:**
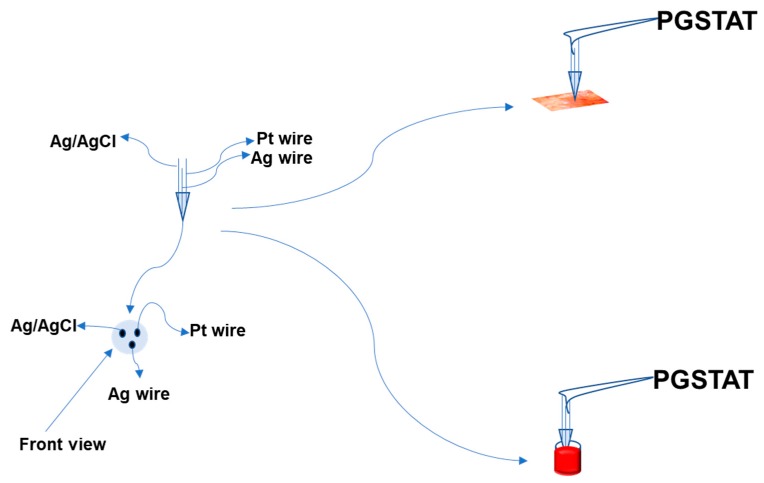
Needle stochastic sensor design, and tissue and whole blood measurements set-up.

For the construction of the three stochastic needle sensors, graphite powder was mixed with paraffin oil until a homogeneous paste was obtained. In order to obtain the modified pastes, solutions of chitosan I, chitosan II, and chitosan III (10^−3^ mol/L) were added to the pastes in a ratio of 1:1 (w/v; mg/µL). Each modified paste was placed in a non-conducting plastic tube (internal diameter 50 µm); a silver wire performed the electric contact between the pastes and the external circuit (Scheme 1).

### 2.4. Stochastic Mode

**Scheme 2:**
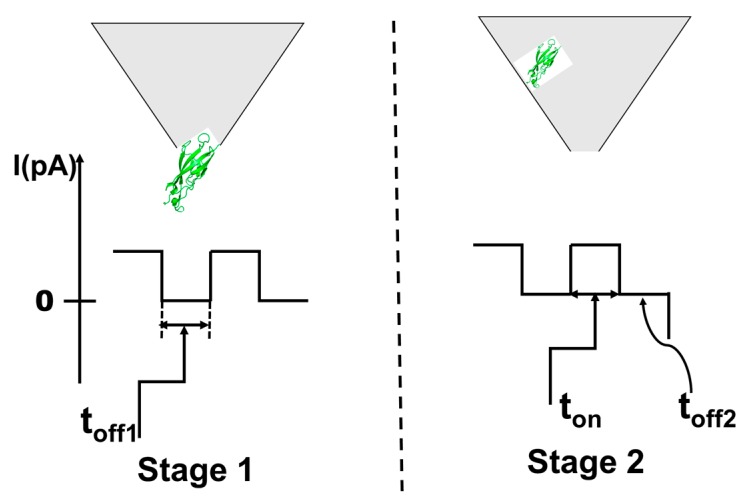
Current development for stochastic sensors, example for the detection of one biomarker.

**Figure 1:**
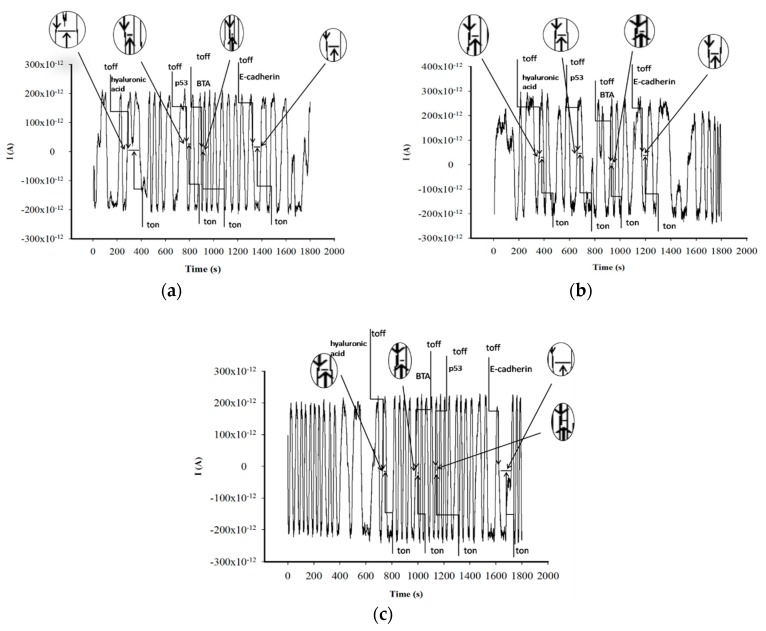
Pattern recognition of p53, E-cadherin, BTA, and hyaluronic acid in whole blood samples using the stochastic needle sensor based on chitosan I/graphite (**a**), chitosan II/graphite (**b**), and chitosan III/graphite (**c**).

**Figure 2:**
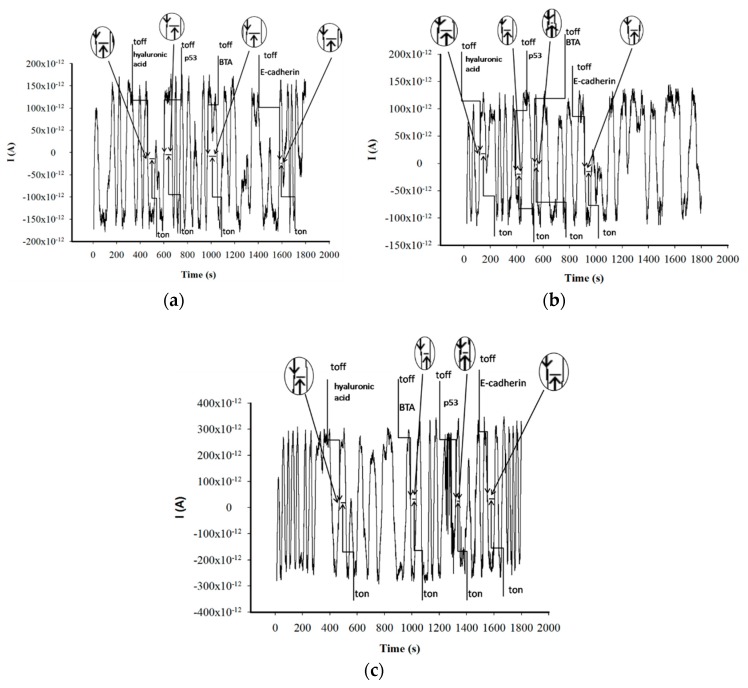
Pattern recognition of p53, E-cadherin, BTA, and hyaluronic acid in tissue samples using the stochastic needle sensor based on chitosan I/graphite (**a**), chitosan II/graphite (**b**), and chitosan III/graphite (**c**).

All measurements were performed using the stochastic mode. The principle of the stochastic needle sensors is based on the channel conductivity. A constant potential of 125 mV was applied and the current was recorded; interval time for the measurements was set to 0.2 s. This method was used for both the qualitative and the quantitative analysis of p53, E-cadherin, BTA, and hyaluronic acid from whole blood and tissue samples of confirmed patients. Two parameters were identified, t_off_ and t_on_, respectively. The t_off_ is known as the signature of the analyte and its value is used for qualitative analysis in pattern recognition. The t_on_ is known as the necessary time of equilibrium for the interaction of the analyte with the wall channel and represents the quantitative parameter (1/t_on_ = a + bxC_biomarker_) (Scheme 2, Figure 1 and Figure 2). The equations of calibration were obtained using the linear regression method.

### 2.5. Samples

Whole blood and tissue samples were received from the Clinic Hospital “Prof. Dr. Th. Burghele” Bucharest. Samples from healthy (10 whole blood samples) and confirmed patients with bladder cancer (whole blood and tissue samples, 30 samples from each) were collected in accordance with the procedures specified in the Ethics Committee Approval no. 11/2013 awarded by the University of Medicine and Pharmacy Carol Davila from Bucharest. Written consent was obtained from all patients. After the samples were collected, immediate analysis was performed. No sample pre-treatment was performed.

## 3. Results and Discussions

### 3.1. Response Characteristics of Stochastic Needle Sensors

The response of the stochastic needle sensors was based on the conductivity of the channel [12,13,14]: the current that flowed through a channel was modified when the analyte entered the channel after a constant potential of 125 mV was applied. The molecular recognition of the analyte of interest took place in two phases. During the first phase, also known as the pattern recognition phase, by applying the potential, the analyte of interest is extracted from the solution into the membrane-solution of the interface, blocking the channel, and the intensity of the channel drops to zero until the whole molecule enters the channel; the time spent on this phase is known as the signature of the analyte (t_off_ value) and its value is used for the qualitative analysis of the analyte of interest (Scheme 2). The signature of the biomarker depends on the size of the biomarker, the geometry of the biomarker; if the biomarker has a 2D or 3D shape (e.g., is protein), the unfolding speed contributes to the value of the signature. According to this, the signature value is cumulating all the responsible time values (size, geometry, unfolding speed), resulting in the time needed to pass from the solution inside the channel. All analytes including biomarkers from a sample going inside the channel are in the order given by their size, shape, and unfolding speed. Therefore, it is difficult to find two analytes that have the same signature when analyzed with the same sensor.

The second phase, known as the binding phase, takes place when the analyte interacts with the wall channel and redox processes take place (Scheme 2). The following equations of equilibrium took place on the binding phases:

Ch(i) + p53(i) ⇔ Ch • p53(i) (1)

Ch(i) + E-cadherin(i) ⇔ Ch • E-cadherin(i)(2)

Ch(i) + BTA (i) ⇔ Ch • BTA (i)(3)

Ch(i) + hyaluronic acid(i) ⇔ Ch • hyaluronic acid(i)(4)

where Ch is the channel and i is the interface. The equilibrium time necessary for the interaction between the analyte and the wall channel, as well as for the redox processes (oxidation or reduction) depends on the molecule analyzed (if it can be oxidized or reduced). According to the redox reaction, the current oscillates between positive and negative values can be defined as t_on_ and it represents the quantitative parameter (Figure 1 and Figure 2). After the redox processes take place, the polarity of the analyte changes and it extracted back into the solution. The value of t_on_ is read in between two t_off_ values (see Scheme 2).

The response characteristics for p53, E-cadherin, BTA, and hyaluronic acid were determined using the t_on_ values and are shown in Table 1. The signatures of the biomarkers (t_off_ values) (p53, E-cadherin, BTA, and hyaluronic acid) were different for the same stochastic needle sensor, indicating that the sensors were selective and that they could be used for the screening tests of whole blood and tissue samples.

The type of chitosan selected (the degree of polymerization) did not influence the limit of determination for E-cadherin, BTA, and hyaluronic acid; in the case of p53, the same limit of determination was obtained when chitosan I and chitosan II were used for the design of the stochastic needle sensors, while a lower limit of determination was obtained when chitosan III was used for the design of the stochastic needle sensor.

The type of chitosan influenced the sensitivity of the measurements: for the assay of p53, the highest sensitivity was obtained for the chitosan I based stochastic needle sensor; for the assay of E-cadherin, the highest sensitivity was obtained for the stochastic needle sensor based on chitosan III; for the assay of BTA, the highest sensitivity was obtained for the chitosan I based stochastic sensor; and for the assay of hyaluronic acid, the highest sensitivity was obtained for the chitosan II based stochastic sensor.

The linear concentration ranges obtained for all stochastic needle sensors covered the asymptomatic patients as well as patients in different stages of bladder cancer. Accordingly, they can be used for screening tests of both asymptomatic and clinically ill patients.

The stochastic needle sensors were used continuously for two years, for the assay of p53, E-cadherin, BTA, and hyaluronic acid. For the two year time period, the variation of their sensitivity was less than 1.00%, indicating their high reliability. Five pastes of the same sensor were made and placed in different plastic tubes; response characteristics of each of the 15 sensors were determined; for the chitosan I based stochastic needle sensor, the sensitivity varied with 0.15%; for the chitosan II based sensor, the sensitivity varied with 0.09%; and for the chitosan III based sensor, the sensitivity varied with 0.10%. These results prove that the design proposed for the sensors is reliable.

The selectivity of the stochastic sensors was determined using the t_off_ values of the biomarkers and other analytes from the biological sample. Different t_off_ values mean selectivity versus those biomarkers/analytes. To date, we could not find the same t_off_ values (signatures) for two biomarkers/analytes, but this does not mean that these sensors are specific. First, the four tested biomarkers had different signatures (Table 1). Carcinoembryonic antigen (CEA), dopamine, HER 1 and HER2 were also tested as possible interfering species, but different signatures were obtained for all of them, proving the selectivity of the proposed stochastic sensors. For the chitosan I based sensor, the signatures obtained were: 3.4 s for CEA, 2.8 s for HER1, and 2.4 s for HER2; for the chitosan II based sensor, the signatures obtained were: 3.2 s for CEA, 2.8 s for HER1, and 2.3 s for HER2; and for the chitosan III based sensor, the signatures obtained were: 3.0 s for CEA, 2.7 s for HER1, and 2.3 s for HER2.

When applying histochemistry for the assay of p53, only a qualitative assay was achieved [19]; limits of detection of 14 pmol/L and 1.29 ng/mL were obtained using an amperometric immunosensor [20] and an amperometric magnetoimmunosensor [21]. For E-cadherin, the best limit of detection obtained to date has been 0.16 ng/mL using an amperometric immunosensor [22], and for BTA, the best limit of determination was 34 U/mL (achieved using immunoassay) [23], while for the assay of hyaluronic acid using a colorimetric enzyme-coupled technique, a limit of detection of 0.3 mg/L was achieved [24]. The proposed stochastic needle sensors had better performances in terms of limits of quantification/determination and sensitivity, and can be used for the simultaneous assay of the four biomarkers.

### 3.2. Analytical Applications

The response characteristics of the stochastic sensors enabled their utilization for the fast screening of whole blood and tissue samples for p53, E-cadherin, BTA, and hyaluronic acid. After recording the diagrams (Figure 1 and Figure 2), the biomarkers were identified in accordance with their signatures (t_off_ values) (Table 1); the corresponding value for t_on_ was read in the diagrams, and the concentration of each biomarker was determined according to the stochastic mode proposed above.

The first step toward validating this method consisted of assaying the biomarkers in whole blood samples obtained from healthy patients, which were spiked with p53, E-cadherin, BTA, and hyaluronic acid.

To prove the accuracy of the method, recovery tests were performed for the assay of p53, E-cadherin, BTA, and hyaluronic acid in spiked samples of whole blood, where known amounts of each biomarker were added, the results of which are shown in Table 2. To perform the recovery test, known quantities of biomarkers were added to whole blood samples and tissue samples; the quantities of the biomarkers were determined before and after the addition of the biomarkers; the difference between the total and initial quantity of the biomarker (the recovered amount) was compared with the quantity of the biomarker added to the sample. As can be seen, recoveries higher than 95.00% were recorded for all biomarkers. Ten whole blood samples from healthy people were analyzed, and the quantities of p53, hyaluronic acid, and E-cadherin were found in the ranges of healthy patients, while BTA was not found in any sample.

Whole blood (50 µL of sample) and tumor tissue samples from patients confirmed with bladder cancer were screened using the proposed stochastic needle sensors and the proposed method. The results are shown in Table 3 and Table 4. Very good correlations were obtained between the values of the determined biomarkers using the stochastic sensors, proving the high reliability of the proposed screening method.

Compared to other methods proposed to date for the assay of these biomarkers, the proposed method is highly reliable, can be used for the simultaneous assay of the proposed biomarkers, is fast, and no sample treatment is needed.

The stochastic sensors were used for more than 100 measurements without refreshing their surfaces and the active surface was washed with distilled water and dried between two measurements of whole blood or tissue samples.

## 4. Conclusions

Three stochastic needle sensors based on graphite paste modified with three types of chitosan: chitosan I (n = 371–744), chitosan II (n = 682–930), and chitosan III (n = 868–1365) were used for the screening of whole blood and tissue samples for four bladder cancer biomarkers. The stochastic needle sensors demonstrated high sensitivity, low limits of determination of these analytes in whole blood and tissue samples, and high reliability when used for the screening of the biological samples.

## Figures and Tables

**Table 1:** The response characteristics of the stochastic sensors used for the assay of p53, E-cadherin, BTA, and hyaluronic acid.

Sensor Based on Graphite Paste and	Signature of the Biomarker t_off_ (s)	Linear Concentration Range (mg/mL)	Equation of Calibration; Correlation Coefficient	Sensitivity (s^−1^/mg mL^−1^)	Limit of Quantification (mg/mL)
p53					
Chitosan I	0.8	1.17 × 10^−12^– 1.00 × 10^−8^	1/t_on_ = 0.04 + 8.88 × 10^8^ × C r = 0.9956	8.88 × 10^8^	1.17 × 10^−12^
Chitosan II	1.2	1.17 × 10^−12^– 1.00 × 10^−8^	1/t_on_ = 0.03 + 2.83 × 10^8^ × C r = 0.9961	2.83 × 10^8^	1.17 × 10^−12^
Chitosan III	1.3	5.28 × 10^−15^– 2.93 × 10^−7^	1/t_on_ = 0.03 + 7.97 × 10^8^ × C r = 0.9998	7.97 × 10^8^	5.28 × 10^−15^
E-Cadherin					
Chitosan I	1.5	8.19 × 10^−13^– 1.00 × 10^−7^	1/t_on_ = 0.01 + 5.88 × 10^9^ × C r = 0.9917	5.88 × 10^9^	8.19 × 10^−13^
Chitosan II	1.8	8.19 × 10^−13^– 1.00 × 10^−8^	1/t_on_ =0.04 + 1.05 × 10^9^ × C r = 0.9900	1.05 × 10^9^	8.19 × 10^−13^
Chitosan III	2.0	8.19 × 10^−13^– 1.00 × 10^−9^	1/t_on_ = 0.01 + 6.08 × 10^9^ × C r = 0.9949	6.08 × 10^9^	8.19 × 10^−13^
BTA					
Chitosan I	1.0	2.00 × 10^−13^– 1.00 × 10^−6^	1/t_on_ = 0.05 + 1.89 × 10^9^ × C r = 0.9999	1.89 × 10^9^	2.00 × 10^−13^
Chitosan II	1.5	2.00 × 10^−13^– 1.00 × 10^−7^	1/t_on_ = 0.02 + 4.62 × 10^8 ×^ C r = 0.9996	4.62 × 10^8^	2.00 × 10^−13^
Chitosan III	1.0	2.00 × 10^−13^– 1.00 × 10^−7^	1/t_on_ = 0.02 + 1.59 × 10^9^ × C r = 0.9935	1.59 × 10^9^	2.00 × 10^−13^
Hyaluronic acid					
Chitosan I	0.5	3.00 × 10^−12^– 1.00 × 10^−9^	1/t_on_ = 0.03 + 5.71 × 10^7^ × C r = 0.9999	5.71 × 10^7^	3.00 × 10^−12^
Chitosan II	1.0	3.00 × 10^−12^– 1.00 × 10^−9^	1/t_on_ = 0.01 + 2.43 × 10^8^ × C r = 0.9999	2.43 × 10^8^	3.00 × 10^−12^
Chitosan III	0.5	3.00 × 10^−12^– 1.00 × 10^−8^	1/t_on_ = 0.03 + 9.67 × 10^7^ × C r = 0.9924	9.67 × 10^7^	3.00 × 10^−12^

**Table 2:** The recovery of p-53, E-cadherin, BTA, and hyaluronic acid in spiked samples using the stochastic needle sensors.

Needle Sensors Based on Graphite Paste and	%, Recovery			
	p53	E-Cadherin	BTA	Hyaluronic Acid
Chitosan l	97.47 ± 0.13	95.06 ± 0.11	99.38 ± 0.10	99.23 ± 0.08
Chitosan ll	99.82 ± 0.10	98.43 ± 0.17	99.69 ± 0.12	99.00 ± 0.15
Chitosan lll	98.49 ± 0.20	97.00 ± 0.24	97.37 ± 0.21	96.52 ± 0.20

**Table 3:** The recovery of p-53, E-cadherin, BTA, and hyaluronic acid in whole blood samples using the stochastic needle sensors (N = 10).

Sample No.	Stochastic Needle Sensors Based on Graphite Paste and	Hyaluronic Acid (pg/mL)	E-Cadherin (pg/mL)	BTA (pg/mL)	p53 (pg/mL)
1	Chitosan I	171.82 ± 0.23	2.99 ± 0.09	14.30 ± 0.12	2.07 ± 0.08
	Chitosan II	123.60 ± 0.15	2.53 ± 0.10	14.78 ± 0.11	2.00 ± 0.08
	Chitosan III	191.00 ± 0.14	2.51 ± 0.08	14.40 ± 0.11	2.78 ± 0.07
2	Chitosan I	196.98 ± 0.18	6.29 ± 0.09	14.64 ± 0.10	2.93 ± 0.07
	Chitosan II	214.00 ± 0.21	6.07 ± 0.08	15.75 ± 0.15	2.97 ± 0.09
	Chitosan III	191.00 ± 0.25	6.46 ± 0.08	14.50 ± 0.12	2.27 ± 0.10
3	Chitosan I	381.03 ± 0.22	8.69 ± 0.07	14.52 ± 0.08	7.40 ± 0.05
	Chitosan II	382.12 ± 0.12	8.89 ± 0.06	14.12 ± 0.09	7.32 ± 0.05
	Chitosan III	357.01 ± 0.17	8.74 ± 0.08	13.27 ± 0.09	7.43 ± 0.06
4	Chitosan I	281.09 ± 0.12	10.02 ± 0.07	43.80 ± 0.13	9.20 ± 0.05
	Chitosan II	298.48 ± 0.17	10.88 ± 0.05	42.45 ± 0.12	9.51 ± 0.09
	Chitosan III	272.02 ± 0.28	10.43 ± 0.09	44.50 ± 0.13	9.94 ± 0.10
5	Chitosan I	228.75 ± 0.25	3.22 ± 0.08	23.10 ± 0.18	6.47 ± 0.03
	Chitosan II	253.03 ± 0.19	3.88 ± 0.05	20.60 ± 0.18	6.40 ± 0.07
	Chitosan III	253.20 ± 0.20	3.06 ± 0.09	20.70 ± 0.12	6.03 ± 0.07
6	Chitosan I	281.01 ± 0.21	6.07 ± 0.08	51.16 ± 0.13	6.39 ± 0.10
	Chitosan II	268.20 ± 0.19	6.49 ± 0.08	51.88 ± 0.13	6.67 ± 0.10
	Chitosan III	255.09 ± 0.22	6.03 ± 0.10	51.30 ± 0.09	6.50 ± 0.08
7	Chitosan I	140.30 ± 0.21	6.29 ± 0.07	64.64 ± 0.25	4.12 ± 0.09
	Chitosan II	123.60 ± 0.15	6.91 ± 0.07	64.13 ± 0.09	4.12 ± 0.03
	Chitosan III	191.00 ± 0.18	6.42 ± 0.09	64.23 ± 0.09	4.10 ± 0.03
8	Chitosan I	380.80 ± 0.27	3.98 ± 0.08	26.35 ± 0.08	4.06 ± 0.08
	Chitosan II	389.82 ± 0.15	3.98 ± 0.08	28.19 ± 0.08	4.18 ± 0.06
	Chitosan III	392.20 ± 0.18	3.23 ± 0.09	27.36 ± 0.07	4.61 ± 0.06
9	Chitosan I	129.40 ± 0.27	4.63 ± 0.08	64.40 ± 0.10	2.42 ± 0.10
	Chitosan II	146.80 ± 0.17	4.00 ± 0.09	64.18 ± 0.10	2.40 ± 0.07
	Chitosan III	154.90 ± 0.23	4.32 ± 0.09	64.07 ± 0.08	2.47 ± 0.07
10	Chitosan I	140.94 ± 0.21	2.88 ± 0.08	14.93 ± 0.07	-*
	Chitosan II	137.27 ± 0.18	3.26 ± 0.10	15.80 ± 0.07	-*
	Chitosan III	153.00 ± 0.20	2.73 ± 0.07	14.40 ± 0.09	- *

* p53 not found.

**Table 4:** The recovery of p-53, E-cadherin, BTA. and hyaluronic acid in tumor tissue samples using the stochastic needle sensors (N = 10).

Sample No.	Stochastic Needle Sensors Based on Graphite Paste and	Hyaluronic Acid (pg/mL)	E-Cadherin (pg/mL)	BTA (pg/mL)	p53 (pg/mL)
1	Chitosan I	281.69 ± 0.12	4.63 ± 0.07	17.40 ± 0.15	4.23 ± 0.10
	Chitosan II	266.00 ± 0.11	4.56 ± 0.07	17.38 ± 0.17	4.34 ± 0.09
	Chitosan III	270.32 ± 0.11	4.69 ± 0.10	17.79 ± 0.10	4.30 ± 0.08
2	Chitosan I	281.79 ± 0.15	7.01 ± 0.08	17.19 ± 0.08	7.75 ± 0.05
	Chitosan II	275.43 ± 0.20	7.44 ± 0.07	17.20 ± 0.12	7.77 ± 0.07
	Chitosan III	279.12 ± 0.23	7.45 ± 0.06	17.31 ± 0.13	7.11 ± 0.08
3	Chitosan I	411.00 ± 0.28	10.20 ± 0.08	28.20 ± 0.09	9.25 ± 0.08
	Chitosan II	411.18 ± 0.24	10.08 ± 0.06	28.19 ± 0.07	9.40 ± 0.03
	Chitosan III	429.20 ± 0.19	10.50 ± 0.06	28.38 ± 0.07	9.40 ± 0.03
4	Chitosan I	437.40 ± 0.11	13.69 ± 0.03	52.15 ± 0.11	10.20 ± 0.07
	Chitosan II	437.00 ± 0.13	13.26 ± 0.04	53.00 ± 0.15	10.13 ± 0.07
	Chitosan III	433.59 ± 0.13	13.42 ± 0.04	52.85 ± 0.13	10.27 ± 0.08
5	Chitosan I	502.90 ± 0.18	6.00 ± 0.07	34.85 ± 0.17	8.32 ± 0.02
	Chitosan II	502.48 ± 0.18	6.59 ± 0.07	34.40 ± 0.15	8.85 ± 0.05
	Chitosan III	502.93 ± 0.23	6.46 ± 0.05	34.21 ± 0.15	8.59 ± 0.03
6	Chitosan I	372.00 ± 0.20	7.56 ± 0.07	80.89 ± 0.18	10.18 ± 0.08
	Chitosan II	351.20 ± 0.20	7.80 ± 0.07	80.65 ± 0.17	10.14 ± 0.06
	Chitosan III	369.17 ± 0.29	7.96 ± 0.08	82.14 ± 0.17	10.21 ± 0.05
7	Chitosan I	218.20 ± 0.18	7.32 ± 0.05	75.45 ± 0.08	5.20 ± 0.10
	Chitosan II	215.10 ± 0.19	7.49 ± 0.03	74.12 ± 0.07	5.66 ± 0.11
	Chitosan III	215.93 ± 0.19	7.01 ± 0.03	77.14 ± 0.07	5.70 ± 0.09
8	Chitosan I	459.20 ± 0.18	6.03 ± 0.05	80.38 ± 0.10	5.32 ± 0.07
	Chitosan II	475.20 ± 0.13	6.49 ± 0.03	80.66 ± 0.10	5.40 ± 0.07
	Chitosan III	457.07 ± 0.13	6.46 ± 0.03	80.69 ± 0.08	5.42 ± 0.04
9	Chitosan I	357.60 ± 0.21	8.00 ± 0.10	72.23 ± 0.09	3.20 ± 0.08
	Chitosan II	385.40 ± 0.24	8.64 ± 0.07	73.00 ± 0.08	3.21 ± 0.09
	Chitosan III	370.42 ± 0.24	8.76 ± 0.07	73.09 ± 0.10	3.15 ± 0.10
10	Chitosan I	412.90 ± 0.20	8.82 ± 0.05	17.82 ± 0.05	- *
	Chitosan II	413.79 ± 0.20	8.89 ± 0.07	17.03±0.07	- *
	Chitosan III	407.86 ± 0.15	8.75 ± 0.06	17.15±0.03	- *

* p53 not found.
